# Mimicking Marker Spread After Disruption of the Blood–Brain Barrier with a Collagen-Based Hydrogel Phantom

**DOI:** 10.3390/biomimetics9110667

**Published:** 2024-11-01

**Authors:** Anastasia S. Vanina, Anastasia I. Lavrova, Dmitry A. Safonov, Alexander V. Sychev, Ivan S. Proskurkin, Eugene B. Postnikov

**Affiliations:** 1 Research Center for Condensed Matter Physics, Kursk State University, Radishcheva St. 33, Kursk 305000, Russia; vanina_as@kursksu.ru (A.S.V.);; 2 Saint-Petersburg State Research Institute of Phthisiopulmonology, Lygovsky Av. 2-4, Saint-Petersburg 191036, Russia; 3 Centre for Nonlinear Chemistry, Immanuel Kant Baltic Federal University, Nevskogo St. 14, Kaliningrad 236041, Russia; 4 Department of Theoretical Physics, Kursk State University, Radishcheva St. 33, Kursk 305000, Russia; 5 Institute of Physics, Saratov State University, Astrakhanskaya St. 83, Saratov 410012, Russia

**Keywords:** blood–brain barrier, parenchyma, microfluidics, phantom studies, Cattaneo equation, hydrogels

## Abstract

Recent studies of the spread of substances penetrating the disrupted blood–brain barrier have revealed that the spread in the parenchyma surrounding a vessel has a complex character. In particular, a flow-like motion occurred for a short time that exhibits a smooth transition to diffusional spread. To address the possible physical background of such behavior, we created a system formed by a hydrogel medium with a channel filled by a marker solution, which can serve as a physical model mimicking the process of a substance passively spreading to the brain’s parenchyma when the blood–brain barrier is disrupted. The key result obtained in this work consists of the conclusion that the above-mentioned two-stage character of the spread process discovered in a previous biophysical experiment on the blood–brain opening in a living mouse may originate from the specificity of transport in porous soft matter with relaxation. We propose a mathematical model based on the extended Cattaneo equation, which reproduces our experimental data; determines the crossover time coinciding with that found in the biological system; and, therefore, provides a means of interpretation of this phenomenon.

## 1. Introduction

The problem of penetration of the blood–brain barrier (BBB) and subsequent spread of substances in the brain’s parenchyma remains a crucial issue with respect to the development of methods of drug delivery with the aim of fighting a wide variety of neurological diseases [1,2]. In addition, leakage through the BBB may accompany microvascular disorders connected to such diseases [3]. Moreover, recent studies have revealed a special role played by the BBB and fluid transport in the paravascular pathway in metabolic waste clearance [4,5], which is important for both the functioning of the brain in its normal physiological state and the prevention of various disorders.

In addition to these ultimate biomedical goals, a separate question is the specificity of the leakage of substances through the disrupted BBB and their subsequent spread into peri- and paravascular spaces and further into the parenchyma. This biophysical issue requires new physical and model insights into the character of transport processes in highly heterogeneous media.

Several experiments carried out in recent years have revealed that such transport can exhibit non-trivial patterns of spread that differ from those of both hydrodynamic and diffusional processes. For example, fluorescent markers fed to the brain’s parenchyma through the disrupted BBB display a transition from fast spread described by the exponential dependence of the spread’s dispersion to a conventional diffusive motion [6]. At least two time-scale dependencies have been detected via the MRI-based registration of a contrast agent spreading into the parenchyma following ultrasound-induced blood–brain barrier (BBB) opening [7]. This phenomenon was later discussed as a possible indication of a more complex Brownian yet non-Gaussian diffusion process that considers stochastically distributed diffusivity in a heterogeneous tissue, which is followed by homogenization at larger spatiotemporal scales [8]. In general, understanding the associated transport process necessitates the development of comprehensive mathematical models that account for the multiple structural, physical, and kinetic processes involved [9,10,11].

For this reason, great interest has emerged recently in the development of phantom media aimed at mimicking the BBB and its surroundings. Such phantoms are intended to create structurally realistic systems with controlled properties that can serve as tests for different (e.g., hydrodynamic or diffusional) models of perfusion through the disrupted barrier. Possible approaches in this direction include the creation of artificial membranes playing the role of the BBB that are biocompatible with endothelial cells used as a biological envelopment; the system, as a whole, can be also immersed in a hydrogel-based scaffold [12], microfluidic systems guiding the respective organoids on chip [13,14], a bioprint of microvasculature seeded by pericytes in an artificial parenchyma [15,16], etc. A more detailed review of materials (among which collagen-based polymer matrices play an important role) and technologies used for this goal can be found in [17]. Another approach is focused on biomimetic artificial media, which are simpler, purely physical constructions, allowing for a more stable quantification of effects of interest and the calibration of experimental setups. Such approaches include, among others, a tubular system for focused ultrasound-induced BBB disruption [18]; the formation of fibril-gel analogs of the microvasculature [19]; the development of technology for the fabrication of a BBB microstructure in a liquid state based on aqueous two-phase printing using alginate–gelatin, alginate–matrigel, and alginate–collage matrices [20], etc.

It should be pointed out that, as briefly mentioned above, this effect, which is our principal interest, is a complex transient character of the outflux from a microvessel to the brain’s parenchyma when the blood–brain barrier is already disrupted. This case does not require exploration of the disruption process itself and, in principle, is close to the problem of modeling infusion and perfusion processes, which can be efficiently reproduced by using hydrogel media formed using components such as gelatin, collagen, etc. [21,22,23]. The main difference consists of the source geometry and conditions on the pressure inducing the initial spread of either fluid or markers.

Recently, we reported the synthesis and investigation of structural and transport properties of a fish collagen-based hydrogel [24,25], which is considered a prospective substance mimicking properties of the brain’s parenchyma. The physicochemical properties of such soft matter can also be adjusted to those of biological tissue through the addition of specific compounds like lipids, surfactants, etc. The relevance of this approach has been demonstrated in the cases of point-source infusions and stroke-like lesions.

The main aim of the present work is to clarify the possible physical premises of the specific short-time transient character of marker spread after the disruption of the BBB, as detected in recent experimental in vivo studies (e.g., [6]). We operate with a hydrogel-based system mimicking a microvessel and its surroundings. The experimental registration of passive transport from this microvessel corresponding to leakage from a disrupted BBB supplied with a corresponding mathematical model should achieve the stated goal in a biomimetic way.

## 2. Materials and Methods

The brain parenchyma phantom used in this study was fabricated from a hydrogel based on marine native collagen derived from the dermal tissues of the African catfish (*Clarias gariepinus*). The detailed procedure for processing the fish skin to extract native collagen has been described in a separate publication [24], where comprehensive information can be found.

**Figure 1:**
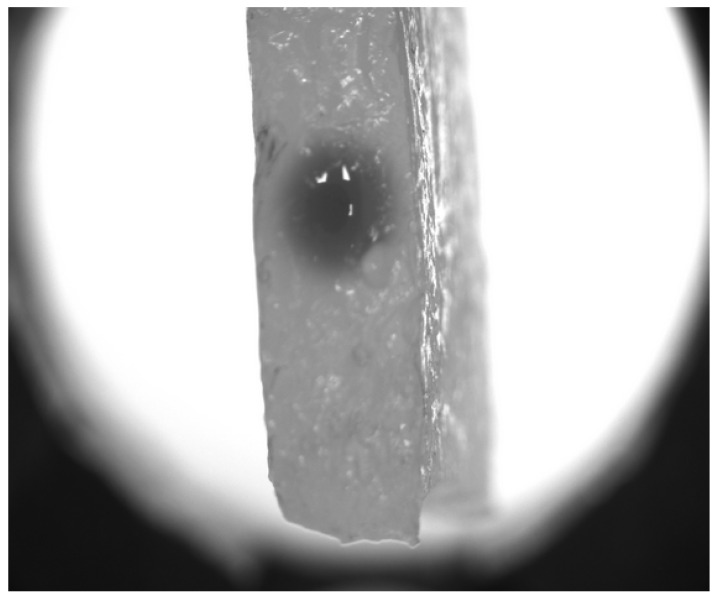
A wide-field photo of the hydrogel with the injected contrast marker after the end of experiment. It is seen that the reconrds were stopped before the moment when the dark spot indicating the marker’s spread reached the boundaries.

To prepare the soft matter-based phantom, the collagen solution with the relative mass concentration of 5% was mixed with glutaraldehyde solution taken in an amount of 10% of the dry collagen’s weight. The addition of the later component plays the role of a cross-linking agent. The obtained mixture was thoroughly mixed, and its volume of 5mL was placed in a beaker and frozen at a temperature of −18 °C for 16 h to facilitate cross-linking. After defrosting, we obtained the piece of a cylinder of hydrogel having a porous microstructure (examples of such microstructure’s pictures obtained by scanning electron microscopy can be found in the earlier paper [24]). A cut of such hydrogel was used for the experiment; see Figure 1.

As a marker substance, we used the water solution of ferroin (chemical formula $C_{36}H_{24}{\mathrm{FeN}}_{62}+$ having the mass concentration 25mM. The choice of this compound was substantiated by its chemical similarity to metal–organic frameworks (MOFs)-based materials conventionally used in biomedical studies utilizing the magnetic resonance imaging as much as possible to penetrate the BBB. At the same time, it can be also considered as a sufficiently opaque dye, which allows for applying optical methods for the registration.

The collagen sample was placed in a Petri dish, and a vertical channel simulating a blood vessel was created using a needle with a diameter of 0.4 mm, which was then filled with a marker substance. Subsequently, the passive leakage of the marker into the surrounding hydrogel was initiated. The experiment was conducted over a time period sufficient to allow the spread of the marker to reach the boundaries of the gel, as shown in Figure 1. Image acquisition was performed from above, allowing the process to be considered in a two-dimensional, axially symmetric geometry without the influence of boundary conditions.

The process was recorded with the stereoscopic microscope Olympus SZX16 (Olympus Corporation, Tokyo, Japan) in the 8-bit grayscale mode as a sequence of images with the 1fps shooting rate, and, simultaneously, as numerical profiles of cross-section going through the spot’s center.

The sequence of images and data were processed with ImageJ (to determine an average intensity in the small vicinity of the spot’s center) and MATLAB software. To establish the correspondence between the concentration of ferroin and the optical density, the background was determined as the average grayscale level of the collagen phantom’s images far from the marker-colored spot, and its value was taken as the zero level. After this, all data were subtracted from this value (since the marker-colored regions are darker, i.e., have lower grayscale values) to obtain a sequence of positive-value profiles. For illustrative purposes, the resulting data were rescaled to the unity at the central region (averaging over 238 pixels surrounding the profile’s maximum).

## 3. Results

### 3.1. Experiment

**Figure 2:**
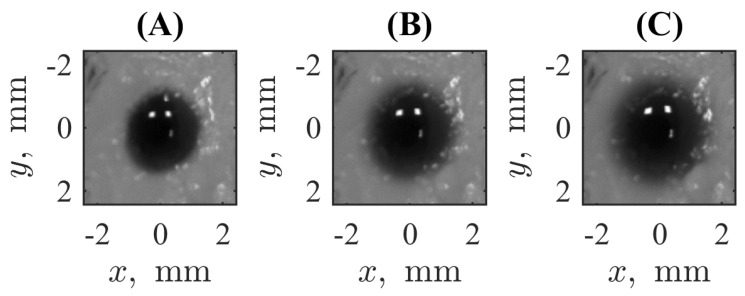
Examples of registered images of the marker’s spread from the channel in the hydrogel. Each of the subpanels shows an instant snapshot taken at the time moments of 0s (**A**), 120s (**B**), and 240s (**C**).

**Figure 3:**
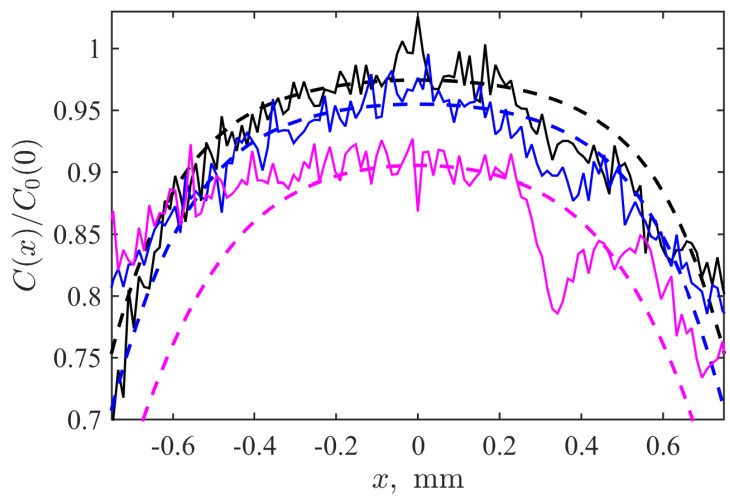
Normed concentration profiles along the diameteral cross-section of the mimicked vessel and its close surrounding recorded experimentally (solid lines) and simulated by the solution of the modified Cattaneo equation (dashed lines). Black, blue and magenta colors correspond to the time moments of 0, 120, and 240 s. Model parameters: $D=4\times 10^{-5}\;{\mathrm{mm}}^{2}/s$, τ=1s, $k=1.5\times 10^{-4}\;s^{-1}$.

Figure 2 shows the pictures qualitatively illustrating the marker spread with time. One can see an explicit elevation of the visible radius of the spot within hundreds of seconds. A more detailed dynamical picture is provided by the Video S1 assembled from the series of images taken from 0s to 540s separated by the time step of 30s. Quantitatively, the central part of the profile is depicted in Figure 3, where solid lines correspond to the cross-sections of the spots presented in Figure 2 and plotted in semi-logarithmic coordinates. Each cross-section passes through the point of symmetry (x=0, y=0) of the axially symmetric picture. These curves allow us to see the radial spread, but we keep the coordinate *x* explicitly to account for fluctuations seen in the real experimental records. One can see both widening the profile respectively to the initial condition and diminishing the magnitude of the central plateau, which indicates that the marker dye initially filling the channel leaves it and goes out to the surrounding hydrogel medium.

The latter fact provides an opportunity to estimate whether this spread has a normal diffusion character or not. Even the analytically solved model in the 2D axially symmetric case with constant initial conditions given by a constant concentration spot of a finite radius *R* results in a complicated integral-based solution. In addition, one can see that real experimental profiles have irregularities. On the contrary, the time evolution of the concentration in the central point possesses a simple analytical solution, which can be rescaled [26] to the form

(1) $$-\frac{1}{\ln\left(1-C\left(0,t\right)/C_{0}\left(0\right)\right)}=\frac{4D}{R^{2}}t$$

which results in the linear dependence on time when the diffusion (with the diffusion coefficient *D*) is normal.

One can see that this sequence of markers follows the linear dependence (highlighted by the dashed straight line) only for large times. Just after the beginning of the process, the observed dependence is significantly nonlinear; the process as a whole is non-stationary and looks like it contains some relaxation before reaching the diffusion-like mode of spread.

Thus, clarification of this issue requires additional studies based on the mathematical modeling of physically reasonable situations. They address the process when the marker dye leaves the fluid-filled cylindrical channel and starts to spread the surrounding complex medium formed by the hydrogel. Regarding the latter, one should keep in mind that it has a biphasic structure and can support relaxation processes.

### 3.2. Mathematical Modeling

In developing the mathematical model describing the experiment, we implement the model, which generalizes the picture of diffusional spread by taking into account its possible finite velocity of spread tightly connected with the initial relaxation processes. The respective fundamental model is well known as the Cattaneo equation (it also can be referenced as the telegraph equation due to the equivalence of their mathematical structure). The original approach [27] has been considered within the context of addressing the limitations of the classical Fourier law by incorporating a time-dependent term that models finite propagation speeds of thermal signals. It combines features of both hyperbolic and parabolic heat conduction models, making it ideal for studying heat transport in materials where thermal waves are present [28] and exhibiting the transition between two qualitatively different short- and long-time behaviors [29]. It also has applications in studying biological tissues. For example, the Cattaneo equation has been used to model the delayed response of heat conduction that provides a framework for modeling time-lagged responses in heat transfer, especially in tissues subjected to hyperthermia treatments or laser heating [30].

At the same time, the Cattaneo equation-based approach and its generalizations attract attention as a suitable tool for modeling several phenomena related to the random walk [31,32] and anomalous diffusive mass transport [33,34] in complex media. However, the majority of works are limited by purely mathematical considerations without real applications to experimental data.

The classic formulation of the Cattaneo (telegraph) equation, respectively, to the concentration *C* reads as

(2) $$\tau \frac{\partial^{2}C}{\partial t^{2}}+\frac{\partial C}{\partial t}=D\nabla^{2}C,$$

where parameter τ is the relaxation time, indicating the delay before concentration changes respond to the applied gradient. The inclusion of the term $\partial^{2}C/\partial t^{2}$ in Equation (2) implies that changes in *C* propagate with a finite speed; *D* is the diffusion coefficient.

**Figure 4:**
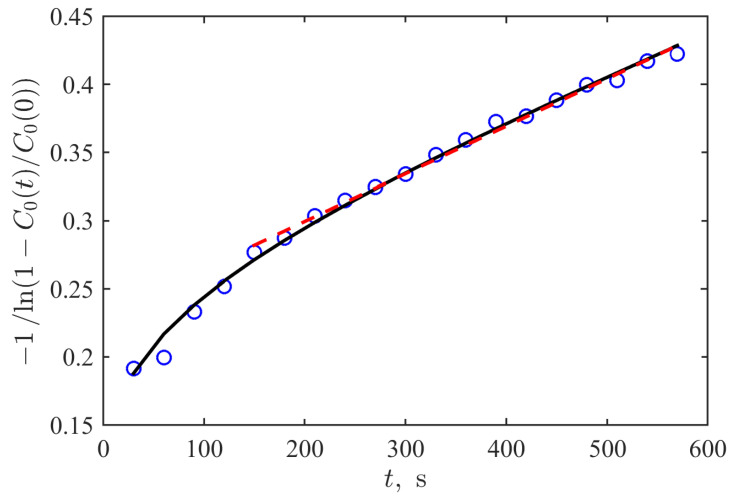
A rescaled representation of the time evolution for the relaxation of the marker concentration in the center of the mimicked vessel obtained in the experiment (circles) and in the simulation with the modified Cattaneo equation (black solid line) with the same parameters as in Figure 3; the red dashed line visually highlights the asymptotic linear dependence.

The carried out simulations with Equation (2) revealed the crossover effect in the transition from the initial spreading of the dye in the experiment to the diffusive regime with a certain delay similar to the experimental one. However, the regime of the diffusion after the crossover region drastically differs from the experimental one; the reproduction of the dependence of the relaxation shown in Figure 4 was not reproducible for the whole time interval.

Based on this observation and taking into account that the collagen sample is a non-uniform structure and the optic method of registration accesses only the thin upper layer of the finite-depth material, we have to modify Equation (2) for our goal. The respective modification is the equation

(3) $$\tau \frac{\partial^{2}C}{\partial t^{2}}+\frac{\partial C}{\partial t}=D\nabla^{2}C-kC,$$

where the parameter *k* accounts for the outflow of the substance either into the bulk of the sample or its “trapping” in the pores of the sample, since the collagen structure is inherently non-uniform.

The simulations with Equation (3) were carried out in the two-dimensional case, i.e.,  the Laplacian was represented as $\nabla^{2}=\partial^{2}/\partial x^{2}+\partial^{2}/\partial y^{2}$, but under conditions of the axially symmetric spread.

It is worth noting that although the “ideal” initial conditions could be stated as a constant within a disk having the radius *R* and zero otherwise ($C\left(x,y,0\right)=C_{0}$ if 0<r<R, C(x,y,0)=0 if r>R), it is preferable to use the smoother version defined as

(4) $$C\left(x,y,0\right)=\frac{C_{0}\left(0\right)}{2}\left(1+\tanh\left[\frac{R^{2}-x^{2}-y^{2}}{s}\right]\right),$$

where the adjustable parameter *s* determines the sharpness of the function’s decay from its maximal value $C_{0}\left(0\right)$ to zero. Such a choice is based on two premises: (i) the Function (4) is differentiable and, as a consequence, the finite-difference scheme implemented for the solution of Equation (3) is more stable; (ii) the actual distribution of the marker in the experiment at the moment corresponding to the initial time of experimental registration also does not have a stepwise shape; see Figure 3. This transient layer is sufficiently narrow and imitates the perivascular space in the biophysical counterpart to the considered model problem. Thus, the value of $s=0.44\;{\mathrm{mm}}^{2}$ was chosen by the fitting of Equation (4) to the experimental distribution shown in Figure 3. Simultaneously, the value R=0.9mm was taken.

The simulations were carried out in the two-dimensional region corresponding to the square with the actual side length of 10mm, i.e., with sides spread from −5 to 5mm; the center of the initial spot correspond to zero coordinates. The model was simulated numerically using the py-pde v. 0.41.0 package in Python, which implements the finite difference method with a fixed computational grid. Spatial discretisation was implemented using a regular orthogonal equispaced grid with an equal number of nodes, N=200, along both axes. The time steps were adaptable during the course of the solution as realized by Python’s numerical algorithm, which was used.

The finite size of the domain used for numerical simulations implies the necessity to state boundary conditions. We defined them as the null-flux conditions on all sides of the square. However, it should be stressed that we examine the dye spreading near the center, covering the radial spread of approximately 0.6–0.8 mm at the maximal time of simulations up to 600s. The spread achieved during such time is far from the square’s sides; i.e., boundary conditions are not expected to play a significant role. Therefore, the results of such short-time simulations can be considered as corresponding to the Cauchy problem on an infinite plane.

**Figure 5:**
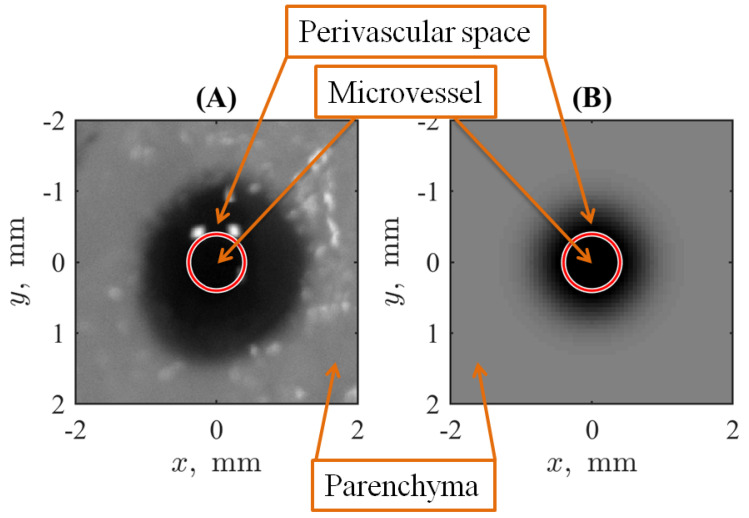
The comparison of the experimental picture of the hydrogel-based system corresponds to the initial stage of the contrast marker spread (**A**) and its counterpart used for mathematical modeling (**B**). For the latter, the zero level of the marker’s concentration is adjusted to the average grayscale level of the part of the photo (**A**) corresponding to the pure hydrogel.

Summarizing, the geometry of numerical simulations in comparison to the experimental biomimetic system and the biological meaning of its regions are shown in Figure 5. Note that the radius of the red circle surrounding the central region denoted as “Microvessel” coincides with the radius of a needle used to form a fluid-filled channel in the hydrogel. In fact, the boundary of the channel is something wider due to the mechanical expansion, and this layer of the collagen structure was soaked with the opaque solution of ferroin when it was introduced into the channel with a needle. However, this layer adjacent to the fluid-filled channel can be considered a kind of imitation of the cellular layer forming the BBB (broken in the actual statement of the problem) and the closest part of the perivascular space in biological systems. This interpretation also supports the usage of a continuously changing Function (4), which defines the initial conditions for simulations. Note also that the dark spot in Figure 5A seems wider than the one in Figure 5B by virtue of the grayscale display in the photographic image. In actual simulations, as shown in Figure 3, the numerical values along the diagonal cross-section of the experimental picture and the mathematical model are well coordinated.

The applied modification of the Cattaneo equation implemented in Equation (3) allowed achieving good quantitative correspondence (the coefficient of determination $R^{2}=0.994$) between results of simulation (the black curve in Figure 4) and the experimental data (circles there). This relaxation curve reproduces the transition between the initial and later stages of the substance spreading. It depicts the first stage with a duration of about 100–150 s as a nonlinear dependence in the used coordinate representation, which originates from the existence of the hyperbolic term in Equation (3) while the second (asymptotic) part is linear that corresponds to the classic diffusive spread. At the same time, it should be noted that the last term in the right-hand side of Equation (3) is crucial for its reproducing, too. Without the leakage, the tangent in the linear region increases more than five times.

We determined the parameters of Equation (3), which provide the mentioned correspondence by the minimization of the absolute deviations between values given its normed solution in the central point at time moments at which the experimental images were taken and the respective values were registered in the real experiment. The values obtained in such a way are listed in the caption to Figure 3. The latter figure allows the exploration of the spatial concentration distribution and its temporal evolution to assess how well the model replicates experimental data under the varying time of the process.

As shown in Figure 3, at the start (the black dashed curve), the concentration profile is the most concentrated near the center, reflecting the initial distribution of the dye. At 120 s, the dye begins to spread, and the peak’s amplitude starts decreasing; however, the reduction is not as rapid, indicating a slight delay. It can be assumed that up to approximately 200 s, a relaxation term plays a significant role. However, with further spreading, diffusion becomes the dominant process, which is somewhat slowed down by a leakage. This is evident at 240 s (see Figure 3, purple dashed and bold lines), where the diffusion process becomes more pronounced, leading to a broader distribution of the dye and a further decrease in the central concentration. Although the experimental curves fluctuate due to the experimental noise and variations in the sample’s structure, the simulated distributions (blue and magenta dashed curves) reasonably reproduce their course on average on the considered radial scales closed to the channel’s surface, i.e., mimicking the processes in paravascular space occurring after the disruption of the blood–brain barrier in the biological case.

## 4. Discussion

A key finding of this study is the distinct transport behavior observed in the proposed system, which comprises a fluid-filled channel embedded in a collagen-based hydrogel. This system facilitates the transport of a marker initially confined within the channel into the surrounding medium. The transport occurs in two stages: an initial non-diffusional phase, which is followed by a second stage characterized by normal axially symmetric diffusion. This two-stage behavior closely mirrors the pattern previously observed in biophysical experiments involving sound-induced opening of the blood–brain barrier in a mouse brain [6]. Moreover, the crossover time, about 150 seconds, determined in this experiment coincides with the same in the biological experiment reported and substantiated in the cited work.

The meaning of this crossover can also be supported by some analytical arguments as follows. It is possible to reduce Equation (3) to a more studied form. For this goal, we represent its solution as the product $C=C(\tilde{r},t)\exp\left(-\alpha t\right)$ and obtain

(5) $$\tilde{\tau }\frac{\partial^{2}\tilde{C}}{\partial t^{2}}+\left(1-2\tau \alpha \right)\frac{\partial \tilde{C}}{\partial t}+\left(\alpha \left(\tau \alpha -1\right)+k\right)\tilde{C}=\tilde{D}\nabla^{2}\tilde{C}.$$

The parameter α should be chosen in such a way that it will compensate the third term in the left-hand side of Equation (5). This property can be achieved when the equality α(τα−1)=−k. This quadratic equation has two roots:

$$\alpha_{1,2}=\frac{1\pm \sqrt{1-4\tau k}}{2\tau }.$$

Taking into account the appropriate values of the parameters found in the numerical simulations as those that allow mimicking the experimental data (τ=1s, $k=1.5\times 10^{-4}\;s^{-1}$), we see that 4τk<<1, and the approximate root fitting the required compensation is α≈k. The respective exponential term describes a very slow time-dependent decay and, therefore, affects only the asymptotic behavior in the diffusive regime reached for large times only. Thus, this substantiates the transition from Equation (2) to Equation (3) to obtain the solution, which fits the experimental data, as explained above.

On the other hand, the space-time-dependent multiplier satisfies the partial differential equation

$$\frac{\tilde{\tau }}{1-2\tau \alpha }\frac{\partial^{2}\tilde{C}}{\partial t^{2}}+\frac{\partial \tilde{C}}{\partial t}=\frac{\tilde{D}}{1-2\tau \alpha }\nabla^{2}\tilde{C},$$

which is the classic Cattaneo (telegraph) Equation (2) with the rescaled coefficients (however, one can even neglect these corrections since 2τα<<1). Properties of its solution are well known [27]. For a short time, the second time derivative dominates, and the solution spreads as a wave-like flow with the velocity $v=\sqrt{D/\tau }$ (it is $6.3\times 10^{-3}\;\mu m/s$). However, at longer timescales, the time derivative of the first order dominates, i.e., the spread is diffusive, and it is characterized not by the velocity but the spreading of the dispersion.

Therefore, the proposed physicochemical system can thus be regarded as a biomimetic model, offering a valuable platform for investigating the physical mechanisms underlying this behavior. In previous work [6], this phenomenon was phenomenologically linked to a non-diffusive, continuous source-driven outflow from the vessel, which abruptly ceases at a specific point in time. However, the factors governing this abrupt transition and the parameters determining the exact timing remain unexplained. In this study, exploring the biomimetic physical system, we can argue that the whole process can be considered a continual one but governed by the generalized Cattaneo equation, which combines different time scales of wave and diffusion in the unified description. Thus, in our view, this result warrants further investigation into which characteristics of biological cells and the extracellular space may give rise to a similar interplay between relaxation and diffusional processes.

**Figure 6:**
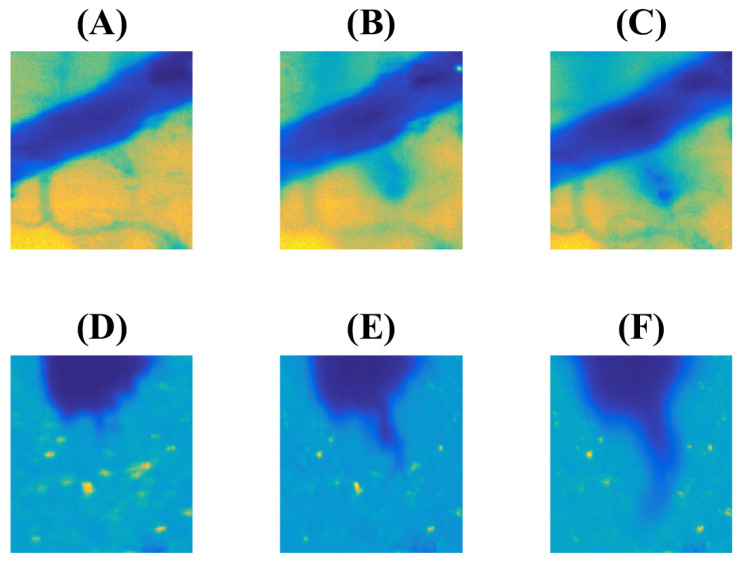
The comparison of the BBB penetration detected in experiment in vivo using the specially reprocessed data obtained in the work [6] (the development of leakage after 42 s (**A**), 100 s (**B**) and 240 s (**C**) from initiating the BBB disruption) and model studies with the hydrogel-based biomimetic physical model (the visibly initiated porecess of leakage (**D**) and its development after 9s (**E**) 31s (**F**)). Enhanced pseudocolors are used to highlight the contrast between the marker solution and its surrounding.

It is worth noting that the diffusion coefficient governing the asymptotic regime of marker spread in the explored hydrogel system was determined by fitting the model curve to the experimental data. This value is comparable to the range of diffusion coefficients typically observed in experiments involving blood–brain barrier (BBB) disruption in living brain tissue. We obtained the value $D=4\times 10^{-5}\;{\mathrm{mm}}^{2}/s$ for the metal-containing marker ferroin. At the same time, the work [7] reports the values, which are in the range of $10÷40\times \;10^{-5}{\mathrm{mm}}^{2}/s$ for the metal-containing contrast media (Dotarem, Gadovist, MultiHance) spreading in the brain of living rat after the ultrasound-induced BBB opening. While the spreading substances exhibit similarities, they are not identical, and there is no perfect correspondence between living tissue and the biomimetic soft matter examined in this study. Nevertheless, the reproducibility of the diffusion coefficient is quite acceptable. We can also mention another example which argues in favor of the developed type of hydrogel-based system as a biomimetic one. The experimental results presented in [6] include, among other findings, the case of asymmetric leakage of a marker into the parenchyma under the influence of intensive ultrasound. Figure 6A–C illustrates three sequential snapshots from one of the isolated events.

To improve visibility, the pseudocolor highlighting of the reciprocal inverse grayscale level intensity squared was applied. As a result, a more intense blue color indicates the higher concentration of the marker, while the yellow background shows the parenchyma. Subpanels (A) and (B) are separated by the time interval 58s; 140s left between images in the subpanels (C) and (B). One can see the emergence of a kind of “protuberance” departing from the main vessel filled with the fluorescence agent.

A qualitatively similar effect can be observed with the considered hydrogel phantom. In this case, we have chosen among various experiments that one, which resulted in a not-so-regular channel formed by a needle during its insertion for creating an analogue to a large vessel. In this case, the combination of communicating pathways between pores of varying sizes within the collagen matrix of the hydrogel led to the formation of a region that facilitated localized leakage rather than the radial leakage observed earlier for the regular channel. The existence of this isolated site of weak penetration leads to the time-dependent picture shown in Figure 6D–E. It is also presented in pseudocolors enhancing the contrast by squaring the grayscale level. The time intervals between images in subpanels (D)–(E) and (E)–(F) of Figure 6 are equal to 9s and 31s. They are shorter than in the case of the biological example due to the difference in media permeability but have similar ratios between their durations. Respectively, the “protuberance developing” in the hydrogel phantom quantitatively resembles the case of a study with a living brain.

## 5. Conclusions

In this work, we reported a physical biomimetic model, which consists of a fluid-filled porous medium (collage-based hydrogel) surrounding a microchannel filled with a contrast agent able to leak into this soft matter and spread there. It is known that the collagen-based hydrogel mimics the principal structural and physicochemical properties of the brain’s parenchyma. Thus, the system imitates the process of substance transport into the brain’s parenchyma when the blood–brain barrier is broken considering the physical effects of the respective transport due to the specificity of the processes in a porous medium.

The experimental results, supported by mathematical modeling, suggest that certain features recently observed in vivo experiments with animal models [6] may arise from the complexity of the soft matter itself rather than from physiological factors. In this context, the key aspect is the reproduction of the two-stage behavior of the speed, which transitions from flow-like dynamics to diffusion. Mathematically, this transition can be described as a solution of the generalized Cattaneo equation, which takes into account both random displacements of the marker and the relaxation of its concentration.

Finally, let us briefly denote the limitations of the mathematical model considered and the prospective directions for future studies in this regard. The spatial scale in Figure 3 demonstrates that the model described by Equation (3), with the parameters provided in the corresponding figure caption, is best suited for capturing the dynamic behavior within the relatively narrow layer surrounding the channel that simulates a blood vessel. It also effectively captures the time-dependent relaxation of concentration within the channel itself (Figure 4). This level of accuracy is sufficient for the primary objective of the present work, which focuses on biomimetic modeling of the processes occurring immediately after the disruption of the blood–brain barrier (BBB). However, the tails of the radial distributions of dye concentration are less accurately reproduced with the far tails being underestimated at longer time scales. It may be related to the specificity of the marker spread in the complex disordered biphasic structure of the hydrogel. Thus, introducing additional mathematical adjustments and taking into account these features can be considered one of the further investigations of the Cattaneo-based mathematical model. At the same time, the reported experimental data can be used for their testing.

## Data Availability

The set of original raw images recorded with the time step 30s are assembled in a video, which is provided as Supplementary Material to this paper. The referenced full experimental images for Figure 6 can be accessed via https://github.com/postnicov/diffmeasure (accessed on 14 September 2024) as a supplement to the paper [6].

## Supplementary Materials

The following supporting information can be downloaded at https://www.mdpi.com/article/10.3390/biomimetics9110667/s1, Video S1: the set of original raw images recorded with the time step 30 s and assembled in a videofile; the grayscale intensity for each frame is denoted there with a colorbar.
